# Preservation of fertility and subsequent childbirth after methotrexate treatment of placenta percreta: a case report

**DOI:** 10.1186/s13256-015-0716-3

**Published:** 2015-10-19

**Authors:** Masato Tamate, Motoki Matsuura, Shutaro Habata, Yushi Akashi, Ryoichi Tanaka, Shinichi Ishioka, Toshiaki Endo, Tsuyoshi Saito

**Affiliations:** Department of Obstetrics and Gynecology, Sapporo Medical University Hospital, South 1, West 16, Chuo-ku, Sapporo, Hokkaido 060-8543 Japan

**Keywords:** Critical care obstetrics, Human chorionic gonadotropin, Methotrexate, Placenta percreta, Preservation of fertility

## Abstract

**Introduction:**

Placenta percreta is associated with maternal morbidity and mortality. A hysterectomy is often needed to control the bleeding in such cases. However, it has been advocated that placenta percreta be managed conservatively to avoid massive pelvic bleeding and to preserve the patient’s fertility. Here, we present a case of placenta percreta diagnosed by magnetic resonance imaging, and treated with systemic administration of methotrexate.

**Case presentation:**

A 27-year-old nulliparous Japanese woman at 39 gestational weeks had an uncomplicated vaginal delivery of a 3244-g infant. However, her placenta was not delivered, and we could not remove it manually. Contrast-enhanced magnetic resonance imaging indicated deep myometrial invasion by placental tissue and the whole placenta was strongly enhanced. Seven days post-partum, her serum human chorionic gonadotropin level was 12,656IU/L. Our patient hoped to preserve her uterus for a future pregnancy. She therefore received 13 courses of methotrexate (50mg/week, intravenous injection). Her serum human chorionic gonadotropin level was undetectable 97 days after the first methotrexate injection. At 117 days post-partum, she had a labor-like pain every three minutes and delivered the placenta. Our patient regained normal menses and at follow-up remained in good health. Two years later, she delivered a healthy daughter.

**Conclusion:**

We should try to detect placenta percreta in high-risk patients by any means. For low-risk patients, we should give a diagnosis swiftly and control any intrauterine infection and massive bleeding.

## Introduction

Placenta percreta, where the chorionic villi invade the myometrium, represents a major cause of obstetric hemorrhage and is associated with maternal morbidity and mortality. A hysterectomy is often needed to control the bleeding in such cases [1]. Even if the bleeding can be controlled, a hysterectomy is often chosen to prevent profuse bleeding and infection during the infant-rearing period. However, it has been advocated that placenta percreta should be managed conservatively to avoid massive pelvic bleeding and preserve the patient’s fertility. Methotrexate (MTX) has been suggested as a possible treatment for placenta percreta to avoid hysterectomy. For such patients, it is also important to diagnose the condition swiftly and not to miss any patients at high risk. Contrast-enhanced magnetic resonance imaging (MRI) technology offers a valuable tool for an early diagnosis and assessment of the treatment outcomes [2]. There has been a paradigm shift in terms of treatment to more conservative methods of management, involving uterine conservation and leaving the placenta in situ with adjuvant treatment of MTX in some cases or simply awaiting spontaneous resorption of the placenta. Here, we present a case of placenta percreta diagnosed by MRI, and treated with systemic administration of MTX.

## Case presentation

Our patient was a 27-year-old Japanese woman, gravida 1 para 0 (G1P0010). Her obstetric history was not significant except for a spontaneous abortion requiring curettage 10 years previously. There was no history of any other pelvic or abdominal surgical intervention.

She was admitted to our hospital with premature rupture of the membranes and had undergone induction of labor at 39 gestational weeks. Two days later, she had an uncomplicated vaginal delivery of a 3244-g male infant with a good Apgar score. However, her placenta was not delivered. We tried manual removal of the placenta, but felt resistance during traction of the umbilical cord. Subsequent Doppler ultrasonography (US) examinations showed a progressive vascular flow to the placental mass in her uterus. We suspected placenta accreta, so we left the whole placenta in her uterus. Fortunately, postpartum bleeding was not severe and the total amount of bleeding was 850mL, even though the umbilical cord was torn during delivery.

**Fig. 1:**
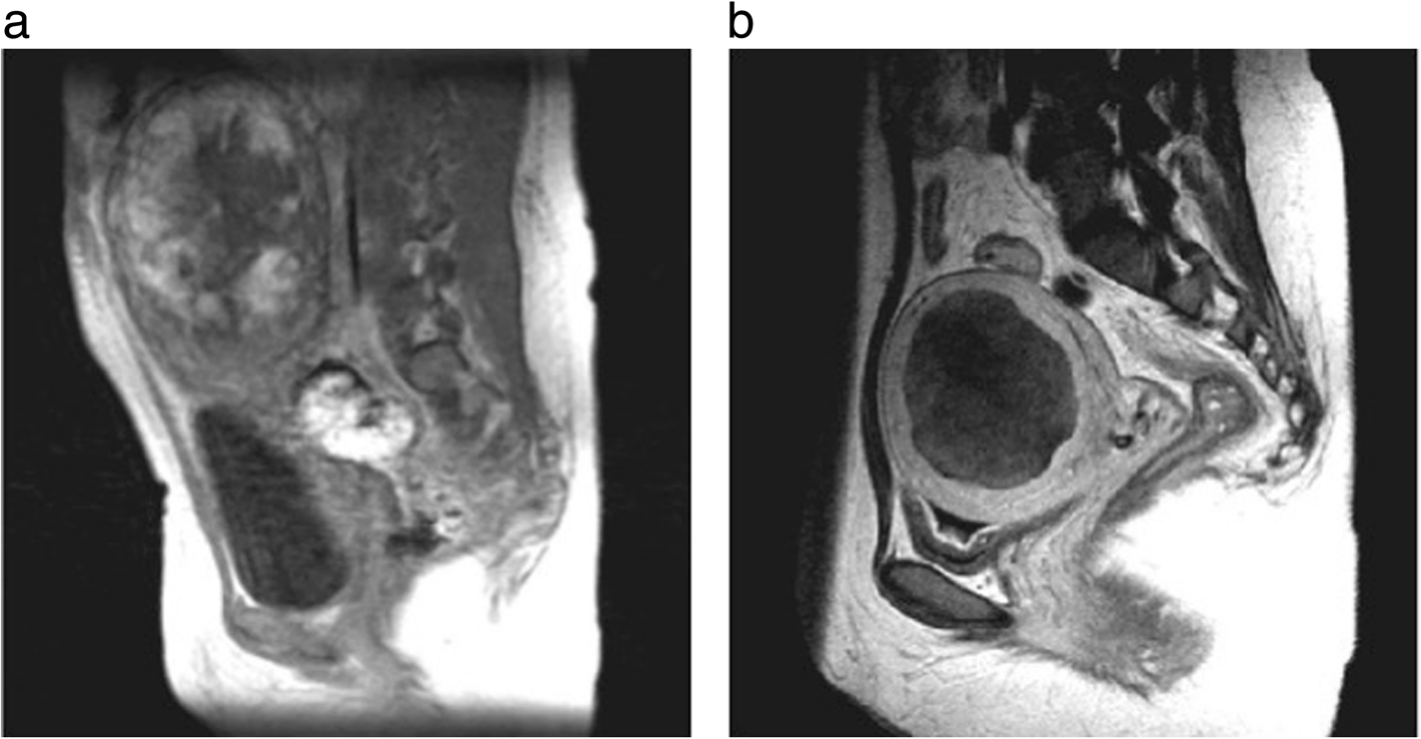
**a** Magnetic resonance imaging scan taken 7 days post-partum. Myometrial invasion by placental tissue is shown and the whole placenta (13.4×11.6×8.6cm) was strongly enhanced. **b** After 13 courses of methotrexate therapy, placental tissue was reduced and none of the placenta (7.0×6.8×6.6cm) was enhanced

We cared for the bleeding and possible infections. At 7 days post-partum, her serum human chorionic gonadotropin (hCG) level was 12,656IU/L. Gadolinium-enhanced MRI also indicated deep myometrial invasion by placental tissues, and the whole placenta was strongly enhanced (Fig. 1a). A general physical examination revealed no abnormalities. Her medical and gynecological histories were unremarkable.

**Fig. 2:**
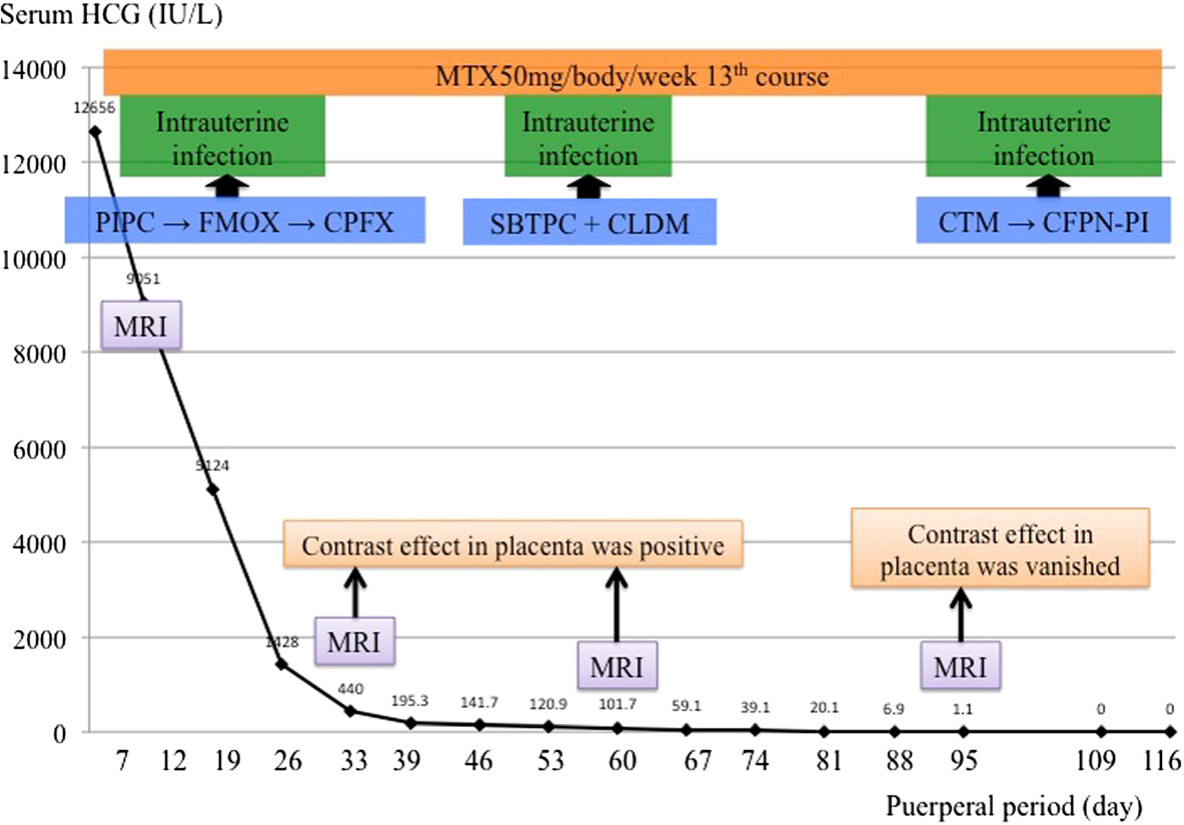
Serum human chorionic gonadotropin (*HCG*) levels were attenuated exponentially by methotrexate (*MTX*) treatment. Our patient had an intrauterine infection, and took three courses of antibiotics. *CLDM* clindamycin, *CPFN-PI* cefcapene pivoxil, *CPFX* ciprofloxacin, *CTM* cefotiam, *FMOX* flomoxef, *MRI* magnetic resonance imaging, *PIPC* piperacillin, *SBTPC* sultamicillin

**Fig. 3:**
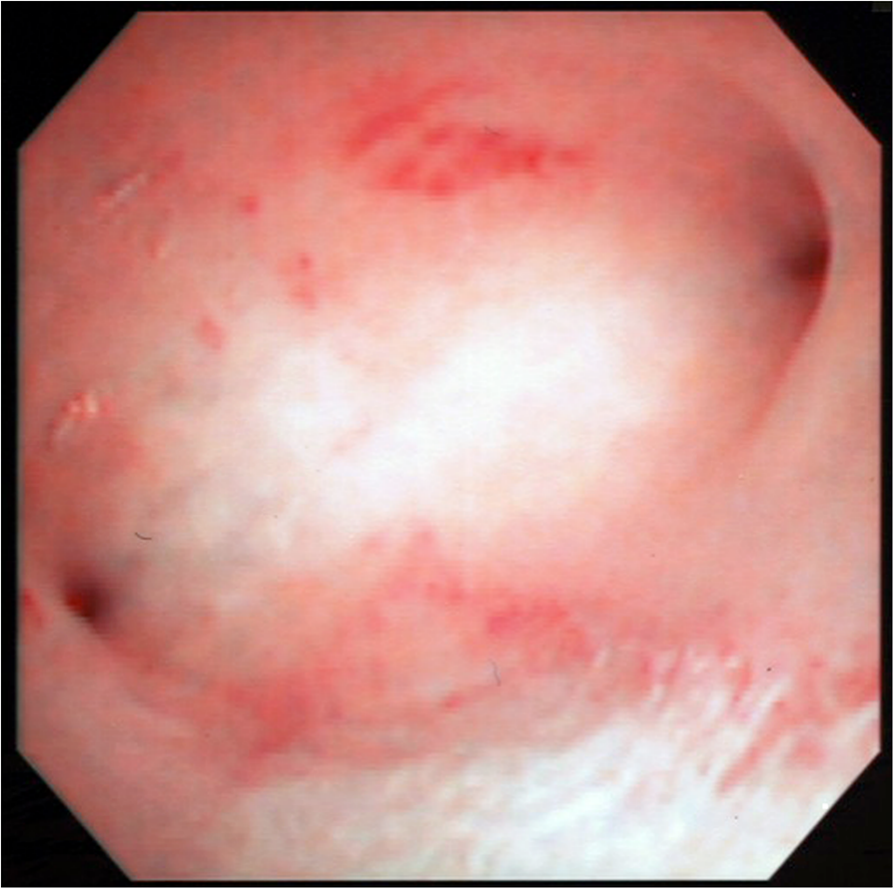
Hysteroscopy image by transvaginal ultrasonography showing a small defect in the endometrium of the posterial wall measuring 8 mm. Another region of the endometrium was defect-free

Our patient hoped to preserve her uterus for a future pregnancy so we decided to administer 50mg MTX by intravenous injection after obtaining informed consent relevant to surgical and other treatment strategies. Hematological, hepatic, and renal functions were evaluated and found to be normal before the MTX injections. Our patient experienced no untoward effects following MTX administration, and her vaginal bleeding diminished gradually over four weeks. To avoid intrauterine manipulation with surgical equipment and subsequent infection (that is, diagnostic hysteroscopy), follow-up MRI using an identical technique was performed every four weeks to assess the treatment response. MTX therapy (50mg/week) was administered 13 times. During the course of this therapy, our patient had an intrauterine infection and took three separate course of antibiotics. Complete resolution of the uterine lesion was confirmed by MRI scans (Fig. 1b). Her serum hCG level was undetectable (<5IU/L) 97 days after the first MTX injection (Fig. 2). At 117 days post-partum, she had a labor-like pain every three minutes and delivered the remnant of the placenta with a little bleeding. The vestigial mass weighed 80g (10×6×4cm) and histopathology indicated placental tissue with necrosis. Her menses resumed 181 days after the first MTX injection, and a follow-up hysteroscopy showed no abnormal findings (Fig. 3).

Two years later, our patient (G2P1011) became pregnant spontaneously. We did not detect any placental lacunae or clear spaces on transabdominal US, nor did we see any depletion of decidua in her uterus on MRI. She underwent an uncomplicated vaginal delivery of a 3304-g female infant with a good Apgar score. Postpartum bleeding was not active and the total bleeding was 910mL. We did not find any thinning of the posterior uterine muscular layer on transabdominal US, and there was no bleeding with the dislodging of the placenta. The placenta had histopathological features of marginal infarction and chorioamnionitis stage 2 by the Blanc category. Two independent pathologists did not note any deficiency in the decidua.

## Discussion

This case illustrates a successful nonsurgical management strategy for placenta percreta utilizing MTX, which resulted in near-complete placental atrophy, as demonstrated by a comparison of pre-treatment and post-treatment MRI scans using gadolinium contrast. In this case, gray-scale US and Doppler imaging were effective for the detection of placental blood flow.

Abnormal placental adherence is believed to result from pathological absence of the normally intervening uterine decidua basalis and Nitabuch’s fibrinoid layer [3, 4]. Superficial invasion of the basalis layer is termed placenta accreta (approximately 75% of cases); deeper invasion of the myometrium is termed placenta increta (approximately 15% of cases); and even deeper invasion involving the serosa or adjacent pelvic organs is termed placenta percreta [5]. This abnormal adherence of the placenta to the uterus can result in catastrophic intrapartum hemorrhage at the time of placental delivery, often necessitating emergency hysterectomy. The prevalence of placenta accreta has increased more than tenfold in the past 30 years to approximately 1 in 2500 deliveries in the USA [6]. This increase appears to relate to the increasing incidence of uterine surgery, which results in a decidual defect that allows abnormal placental ingrowth [7]. However, our patient did not have this risk factor. It is also possible that placenta percreta is associated with an abnormal position of implantation in early pregnancy, for example, cornual and angular pregnancies [8].

Placenta percreta is associated with considerable maternal morbidity and mortality. Indeed, most cases of placenta percreta require a hysterectomy to achieve adequate hemostasis [9]. Although a hysterectomy remains the definitive therapy for placenta increta, some women (such as in our case) desire conservation of the uterus to ensure future fertility. For this reason, less-extreme surgical approaches, including wedge resection of the affected myometrium, have been proposed as a method of permitting subsequent pregnancy. MTX is a potent antimetabolite, with mechanisms of action that include inhibition of trophoblast cells, reduction of placental neovascularization, and attenuation of placental growth factors [10]. In this case, MTX was effective in reducing the blood flow in her placenta diagnosed by MRI and US. Placenta previa and prior intrauterine manipulation have been identified as significant synergistic factors for the development of placenta accreta or percreta, with rates as high as 50–67% in patients with a combination of more than two prior Cesarean deliveries and a placenta previa [11]. However, our patient did not have a history of prior Cesarean delivery.

MRI and US are effective diagnostic tools. The overall sensitivity and specificity of US for the diagnosis of placenta accreta have been reported to be 77–93% and 71–96%, respectively [11]. The US features of placenta accreta include loss of the normal retroplacental clear space, anomalies of the bladder–myometrium interface, prominent placental lacunae, and increased vascularity at the interface of the uterus and bladder [11, 12]. By contrast, the overall sensitivity and specificity of MRI have been reported as 80–88% and 65–100%, respectively. MRI features considered diagnostic of placenta accreta include abnormal uterine bulging, heterogeneous placental signal intensity on T2-weighted images, and the presence of dark intraplacental bands associated with lacunae on such images [11, 12]. In our case, MRI in puerperium showed the absence of dark intraplacental bands.

Our patient had risk factors for placental abnormality, including a previous abortion, and dilatation and curettage. However, if dilatation and curettage become a risk of placenta accreta, she may have a repeat placenta accreta. Previous placenta previa, submucosal fibroids, prior Cesarean delivery, and multiparity are other recognized risk factors for placenta increta and percreta. Recent studies have shown that patients with placenta accreta or percreta have high serum alpha-fetoprotein and serum hCG levels.

It took 117 days from the initial delivery for our patient to achieve a satisfactory therapeutic response to MTX, shown by her declining serum hCG levels and confirmed by contrast-enhanced MRI. This successful response obviated the need for diagnostic hysteroscopy and possible hysterectomy to remove the residual placenta.

Placental invasion of the myometrium may be difficult to discern using routine US. Accordingly, use of contrast-enhanced MRI to confirm any abnormal placentation warrants consideration in such clinical settings [13].

## Conclusions

We should try to detect placenta accreta and percreta in high-risk cases using US and MRI. For low-risk cases, we should give a diagnosis swiftly and control any intrauterine infection and massive bleeding. Early diagnosis is important so that the patient can be prepared and adequately counselled with regard to the treatment options and their possible consequences. Fever may also represent an inflammatory response to tissue necrosis in the absence of any infectious source. Infectious morbidity can be reduced by use of prophylactic broad-spectrum antibiotic therapy [14] and the patient in our case had a successful subsequent live delivery after placenta percreta.

## Consent

Written informed consent was obtained from the patient for publication of this case and accompanying images. A copy of the written consent is available for review by the Editor-in- Chief of this journal.

## Abbreviations

hCG: human chorionic gonadotropin
MRI: magnetic resonance imaging
MTX: methotrexate
US: ultrasonography
